# Genome-Wide Association Study of Susceptibility to Idiopathic Pulmonary Fibrosis

**DOI:** 10.1164/rccm.201905-1017OC

**Published:** 2019-11-11

**Authors:** Richard J. Allen, Beatriz Guillen-Guio, Justin M. Oldham, Shwu-Fan Ma, Amy Dressen, Megan L. Paynton, Luke M. Kraven, Ma'en Obeidat, Xuan Li, Michael Ng, Rebecca Braybrooke, Maria Molina-Molina, Brian D. Hobbs, Rachel K. Putman, Phuwanat Sakornsakolpat, Helen L. Booth, William A. Fahy, Simon P. Hart, Mike R. Hill, Nik Hirani, Richard B. Hubbard, Robin J. McAnulty, Ann B. Millar, Vidyia Navaratnam, Eunice Oballa, Helen Parfrey, Gauri Saini, Moira K. B. Whyte, Yingze Zhang, Naftali Kaminski, Ayodeji Adegunsoye, Mary E. Strek, Margaret Neighbors, Xuting R. Sheng, Gunnar Gudmundsson, Vilmundur Gudnason, Hiroto Hatabu, David J. Lederer, Ani Manichaikul, John D. Newell, George T. O’Connor, Victor E. Ortega, Hanfei Xu, Tasha E. Fingerlin, Yohan Bossé, Ke Hao, Philippe Joubert, David C. Nickle, Don D. Sin, Wim Timens, Dominic Furniss, Andrew P. Morris, Krina T. Zondervan, Ian P. Hall, Ian Sayers, Martin D. Tobin, Toby M. Maher, Michael H. Cho, Gary M. Hunninghake, David A. Schwartz, Brian L. Yaspan, Philip L. Molyneaux, Carlos Flores, Imre Noth, R. Gisli Jenkins, Louise V. Wain

**Affiliations:** ^1^ Department of Health Sciences, University of Leicester, Leicester, United Kingdom; ^2^ Unidad de Investigacion, Hospital Universitario Ntra. Sra. de Candelaria and; ^64^ Instituto de Tecnologías Biomédicas, Universidad de La Laguna, Santa Cruz de Tenerife, Spain; ^3^ Department of Internal Medicine, University of California Davis, Davis, California; ^4^ Division of Pulmonary and Critical Care Medicine; ^36^ Center for Public Health Genomics, and; ^37^ Department of Public Health Sciences, University of Virginia, Charlottesville, Virginia; ^5^ Genentech, South San Francisco, California; ^6^ The University of British Columbia Centre for Heart Lung Innovation, St. Paul’s Hospital, Vancouver, British Columbia, Canada; ^7^ Nuffield Department of Orthopaedics, Rheumatology and Musculoskeletal Sciences; ^19^ Clinical Trial Service Unit and Epidemiological Studies Unit, Nuffield Department of Population Health; ^53^ Wellcome Centre for Human Genetics, and; ^56^ Oxford Endometriosis Care and Research Centre, Nuffield Department of Women’s and Reproductive Health, University of Oxford, Oxford, United Kingdom; ^8^ Division of Epidemiology and Public Health and; ^57^ Division of Respiratory Medicine, University of Nottingham, Nottingham, United Kingdom; ^9^ National Institute for Health Research, Nottingham Biomedical Research Centre and; ^24^ Respiratory Medicine, Nottingham University Hospitals NHS Trust, Nottingham, United Kingdom; ^10^ Servei de Pneumologia, Laboratori de Pneumologia Experimental, Instituto de Investigación Biomédica de Bellvitge (IDIBELL), Barcelona, Spain; ^11^ Campus de Bellvitge, Universitat de Barcelona, Barcelona, Spain; ^12^ Centro de Investigación Biomédica en Red de Enfermedades Respiratorias, Instituto de Salud Carlos III, Madrid, Spain; ^13^ Channing Division of Network Medicine; ^14^ Division of Pulmonary and Critical Care Medicine; ^32^ Department of Radiology, and; ^33^ Center for Pulmonary Functional Imaging, Brigham and Women’s Hospital, Boston, Massachusetts; ^15^ Department of Medicine, Faculty of Medicine Siriraj Hospital, Mahidol University, Bangkok, Thailand; ^16^ Department of Thoracic Medicine, University College London Hospitals NHS Foundation Trust, London, United Kingdom; ^17^ Discovery Medicine, GlaxoSmithKline, Stevenage, United Kingdom; ^18^ Respiratory Research Group, Hull York Medical School, Castle Hill Hospital, Cottingham, United Kingdom; ^20^ Medical Research Council Centre for Inflammation Research, The University of Edinburgh, Edinburgh, United Kingdom; ^21^ UCL Respiratory Centre for Inflammation and Tissue Repair, University College London, London, United Kingdom; ^22^ Academic Respiratory Unit, School of Clinical Sciences, University of Bristol, Bristol, United Kingdom; ^23^ Cambridge Interstitial Lung Disease Service, Royal Papworth Hospital, Cambridge, United Kingdom; ^25^ Division of Pulmonary, Allergy and Critical Care Medicine and; ^26^ Simmons Center for Interstitial Lung Diseases, University of Pittsburgh, Pittsburgh, Pennsylvania; ^27^ Section of Pulmonary, Critical Care and Sleep Medicine, Yale School of Medicine, New Haven, Connecticut; ^28^ Section of Pulmonary and Critical Care, Department of Medicine, The University of Chicago, Chicago, Illinois; ^29^ Department of Respiratory Medicine, Landspital University Hospital, Reykjavik, Iceland; ^30^ Faculty of Medicine University of Iceland, Reykjavik, Iceland; ^31^ Icelandic Heart Association, Kopavogur, Iceland; ^34^ Department of Medicine, College of Physicians and Surgeons and; ^35^ Department of Epidemiology, Mailman School of Public Health, Columbia University, New York, New York; ^38^ Division of Cardiovascular and Pulmonary Imaging, Department of Radiology, University of Iowa Carver College of Medicine, Iowa City, Iowa; ^39^ Department of Radiology, University of Washington, Seattle, Washington; ^40^ Department of Medicine, Pulmonary Center, Boston University, Boston, Massachusetts; ^41^ NHLBI’s Framingham Heart Study, Framingham, Massachusetts; ^42^ Center for Precision Medicine, Department of Internal Medicine, Wake Forest School of Medicine, Winston-Salem, North Carolina; ^43^ Department of Biostatistics, Boston University School of Public Health, Boston, Massachusetts; ^44^ Center for Genes, Environment and Health, National Jewish Health, Denver, Colarado; ^45^ Department of Biostatistics and Informatics; ^61^ Department of Medicine, and; ^62^ Department of Immunology, University of Colorado Denver, Denver, Colorado; ^46^ Institut universitaire de cardiologie et de pneumologie de Québec, Université Laval, Québec, Québec, Canada; ^47^ Department of Genetics and Genomic Sciences and; ^48^ Icahn Institute for Data Science and Genomic Technology, Icahn School of Medicine at Mount Sinai, New York, New York; ^49^ Merck Research Laboratories, Genetics and Pharmacogenomics, Boston, Massachusetts; ^50^ Respiratory Division, Department of Medicine, University of British Columbia, Vancouver, British Columbia, Canada; ^51^ University Medical Center Groningen, University of Groningen, Department of Pathology and Medical Biology and; ^52^ Groningen Research Institute for Asthma and COPD, Groningen, the Netherlands; ^54^ Department of Biostatistics, University of Liverpool, Liverpool, United Kingdom; ^55^ Division of Musculoskeletal and Dermatological Sciences, University of Manchester, Manchester, United Kingdom; ^58^ National Institute for Health Research, Leicester Respiratory Biomedical Research Centre, Glenfield Hospital, Leicester, United Kingdom; ^59^ National Institute for Health Research Respiratory Clinical Research Facility, Royal Brompton Hospital, London, United Kingdom; ^60^ National Heart and Lung Institute, Imperial College, London, United Kingdom; and; ^63^ Instituto Tecnológico y de Energías Renovables, S.A., Santa Cruz de Tenerife, Spain

**Keywords:** genetics, epidemiology, KIF15, MAD1L1, DEPTOR

## Abstract

**Rationale:**

Idiopathic pulmonary fibrosis (IPF) is a complex lung disease characterized by scarring of the lung that is believed to result from an atypical response to injury of the epithelium. Genome-wide association studies have reported signals of association implicating multiple pathways including host defense, telomere maintenance, signaling, and cell–cell adhesion.

**Objectives:**

To improve our understanding of factors that increase IPF susceptibility by identifying previously unreported genetic associations.

**Methods:**

We conducted genome-wide analyses across three independent studies and meta-analyzed these results to generate the largest genome-wide association study of IPF to date (2,668 IPF cases and 8,591 controls). We performed replication in two independent studies (1,456 IPF cases and 11,874 controls) and functional analyses (including statistical fine-mapping, investigations into gene expression, and testing for enrichment of IPF susceptibility signals in regulatory regions) to determine putatively causal genes. Polygenic risk scores were used to assess the collective effect of variants not reported as associated with IPF.

**Measurements and Main Results:**

We identified and replicated three new genome-wide significant (*P* < 5 × 10^−8^) signals of association with IPF susceptibility (associated with altered gene expression of *KIF15*, *MAD1L1*, and *DEPTOR*) and confirmed associations at 11 previously reported loci. Polygenic risk score analyses showed that the combined effect of many thousands of as yet unreported IPF susceptibility variants contribute to IPF susceptibility.

**Conclusions:**

The observation that decreased *DEPTOR* expression associates with increased susceptibility to IPF supports recent studies demonstrating the importance of mTOR signaling in lung fibrosis. New signals of association implicating *KIF15* and *MAD1L1* suggest a possible role of mitotic spindle-assembly genes in IPF susceptibility.

**At a Glance Commentary**

**Scientific Knowledge on the Subject**

Idiopathic pulmonary fibrosis (IPF) is a devastating disease where the lungs become scarred. It is not known what causes the scarring, but there have been 17 regions of the genome that have been reported as associated with increased susceptibility to IPF from previous genome-wide association studies. These identify host defense (particularly mucus production), cell–cell adhesion, signaling, and telomere maintenance as important processes in the development of lung fibrosis.

**What This Study Adds to the Field**

By combining all previous IPF genome-wide association studies, we have identified three novel regions of the genome identified with IPF risk and confirmed 11 of the 17 previously reported regions. The three novel regions implicate the genes DEPTOR, KIF15, and MAD1L1. These findings support recent research that shows mTOR signaling promotes lung fibrogenesis and also implicate spindle-assembly genes in the development of IPF.

Idiopathic pulmonary fibrosis (IPF) is a devastating lung disease characterized by the buildup of scar tissue. It is believed that damage to the alveolar epithelium is followed by an aberrant wound-healing response leading to the deposition of dense fibrotic tissue, reducing the lungs’ flexibility and inhibiting gas transfer (1). Treatment options are limited, and half of individuals diagnosed with IPF die within 3 to 5 years (1, 2). Two drugs (pirfenidone and nintedanib) have been approved for the treatment of IPF, but neither offer a cure, and they only slow disease progression.

IPF is associated with a number of environmental and genetic factors. Identifying regions of the genome contributing to disease risk improves our understanding of the biological processes underlying IPF and helps in the development of new treatments (3). To date, genome-wide association studies (4–8) (GWAS) have reported 17 common variant (minor allele frequency [MAF] >5%) signals associated with IPF, stressing the importance of host defense, telomere maintenance, cell–cell adhesion, and signaling with respect to disease susceptibility. The sentinel (most strongly associated) variant, rs35705950, in one of these signals that maps to the promoter region of the *MUC5B* gene has a much larger effect on disease susceptibility than other reported risk variants with each copy of the risk allele associated with a fivefold increase in odds of disease (9). Despite this, the variant rs35705950 has a risk allele frequency of only 35% in cases (compared with 11% in the general population) and so does not explain all IPF risk. Rare variants (MAF < 1%) in telomere-related and surfactant genes have also been implicated in familial pulmonary fibrosis and sporadic IPF (10, 11).

In this study, we aimed to identify previously unreported genetic associations with IPF to improve our understanding of disease susceptibility and generate new hypotheses about disease pathogenesis. We conducted a large GWAS of IPF susceptibility by utilizing all European cases and controls recruited to any previously reported IPF GWAS (5–8) and meta-analyzing the results. This was followed by replication in individuals not previously included in IPF GWAS and bioinformatic analysis of gene expression data to identify the genes underlying the identified association signals. As specific IPF-associated variants have also been shown to overlap with other related respiratory traits including lung function in the general population, chronic obstructive pulmonary disease (COPD) (with genetic effects in opposite directions between COPD and IPF) (12–14), and interstitial lung abnormalities (ILAs) (which might be a precursor lesion for IPF) (15), we tested for association of the IPF susceptibility variants with these respiratory phenotypes in independent datasets. Finally, using polygenic risk scores, we tested whether there was still a substantial contribution to IPF risk from genetic variants with as yet unconfirmed associations with IPF susceptibility.

Some of the results of these studies have been previously reported in the form of two abstracts and a preprint (16–18).

## Methods

### Study Cohorts

We analyzed genome-wide data from three previously described independent IPF case–control collections (named here as the Chicago [5], Colorado [6], and UK [8] studies; please refer to the online supplement for summaries of these collections). Two more independent case–control collections (named here as the UUS [United States, United Kingdom, and Spain] and Genentech studies) were included as replication datasets. The new UUS study recruited cases from the United States, United Kingdom, and Spain and selected controls from UK Biobank (19) (full details on the recruitment, genotyping, and quality control of UUS cases and controls can be found in the online supplement). The previously described (20) Genentech study consisted of cases from three IPF clinical trials and controls from four non-IPF clinical trials (*see* the online supplement). All studies were restricted to unrelated individuals of European ancestry, and we applied stringent quality control measures (full details of the quality control measures of each study can be found in the online supplement and Figure E1 in the online supplement). All studies diagnosed cases using American Thoracic Society and European Respiratory Society guidelines (21–23) and had appropriate institutional review board or ethics approval.

Genotype data for the Colorado, Chicago, UK, and UUS studies were imputed separately using the Haplotype Reference Consortium r1.1 panel (24) (*see* the online supplement). For individuals in the Genentech study, genotypes were derived from whole-genome sequencing data. Duplicated individuals between studies were removed (*see* the online supplement).

### Identification of IPF Susceptibility Signals

In each of the Chicago, Colorado, and UK studies separately, a genome-wide analysis of IPF susceptibility, using SNPTEST (25) v2.5.2, was conducted adjusting for the first 10 principal components to account for fine-scale population structure. Only biallelic autosomal variants that had a minor allele count ≥10 were in the Hardy–Weinberg Equilibrium (*P* > 1 × 10^−6^) and were well-imputed (imputation quality *R*^2^ > 0.5) in at least two studies were included. A genome-wide meta-analysis of the association summary statistics was performed across the Chicago, Colorado, and UK studies using R v3.5.1 (discovery stage). Conditional analyses were performed to identify independent association signals in each locus (*see* the online supplement).

Sentinel variants (defined as the variant in an association signal where no other variants within 1 Mb showed a stronger association) of the novel signals reaching genome-wide significance in the meta-analysis (*P* < 5 × 10^−8^), and nominally significant (*P* < 0.05) with consistent direction of effect in each study, were further tested in the replication samples. We considered novel signals to be associated with IPF susceptibility if they reached a Bonferroni-corrected threshold (*P* < 0.05/number of signals followed up) in a meta-analysis of the UUS and Genentech studies (replication stage; *see* the online supplement). Previously reported signals with *P* < 5 × 10^−8^ in the discovery meta-analysis were deemed a confirmed association.

### Characterization of Signals and Functional Effects

To further refine our association signals to include only variants with the highest probabilities of being causal, Bayesian fine-mapping was undertaken. This approach takes all variants within the associated locus and, using the GWAS association results, calculates the probability of each variant being the true causal variant (under the assumptions that there is one causal variant and that the causal variant has been measured). The probabilities are then combined across variants to define the smallest set of variants that is 95% likely to contain the causal variant (i.e., the 95% credible set) for each IPF susceptibility signal (*see* the online supplement).

To identify which genes might be implicated by the IPF susceptibility signals, we identified whether any variants in the credible sets were genic coding variants and defined as deleterious (using Variant Effect Predictor [VEP] [26]). In addition, we tested to see if any of the credible set variants were associated with gene expression using three expression quantitative trait loci (eQTL) resources (the Lung eQTL study [*n* = 1,111] [27–29], the NESDA-NTR [Netherlands Study of Depression and Anxiety-Netherlands Twin Register] blood eQTL database [*n* = 4,896] [30], and 48 tissues in GTEx [31] [*n* between 80 and 491]; *see* the online supplement). Where IPF susceptibility variants were found to be associated with expression levels of a gene, we tested whether the same variant was likely to be causal both for differences in gene expression and IPF susceptibility. We only report associations with gene expression where the probability of the same variant driving both the IPF susceptibility signal and gene expression signal exceeded 80% (*see* the online supplement).

To investigate whether the IPF susceptibility variants that were in noncoding regions of the genome might be in regions with regulatory functions (for example, in regions of open chromatin), we investigated the likely functional impact of those variants using DeepSEA (deep learning-based sequence analyzer) (32). Taking all of the IPF susceptibility variants together, we tested for overall enrichment in regulatory regions specific to particular cell and tissue types using FORGE (functional element overlap analysis of the results of GWAS experiments) (33) and GARFIELD (GWAS analysis of regulatory or functional information enrichment with LD correction) (34). Finally, we investigated whether the genes that were near to the IPF susceptibility variants were more likely to be differentially expressed between IPF cases and controls in four lung epithelial cell types, using SNPsea (35). More details are provided in the online supplement.

### Shared Genetic Susceptibility with Other Respiratory Traits

As previous studies have reported shared genetic susceptibility for IPF and other lung traits (12, 13, 15), we investigated whether the new and previously reported IPF susceptibility signals were associated with quantitative lung function measures in a GWAS of 400,102 individuals (36) or with ILAs in a GWAS comparing 1,699 individuals with an ILA and 10,247 controls (37). Lung function measures investigated were FEV_1_, FVC, the ratio FEV_1_/FVC (used in the diagnosis of COPD), and peak expiratory flow. We applied a Bonferroni corrected *P* value threshold to define variants also associated with ILAs or lung function.

### Polygenic Risk Scores

The contribution of as yet unreported variants to IPF susceptibility was assessed using polygenic risk scores. For each individual in the UUS study, the weighted score was calculated as the number of risk alleles, multiplied by the effect size of the variant (as a weighting), summed across all variants included in the score. Effect sizes were taken from the discovery GWAS and independent variants selected using a linkage disequilibrium *r*^2^ ≤ 0.1. As we wanted to explore the contribution from as yet unreported variants, we excluded variants within 1 Mb of each IPF susceptibility locus from the risk score calculation (*see* the online supplement).

The score was tested to identify whether it was associated with IPF susceptibility, adjusting for 10 principal components to account for fine-scale population structure, using PRSice v1.25 (38). We altered the number of variants included in the risk score calculation using a sliding *P* threshold (*P*_T_) such that the variant had to have a *P* value <*P*_T_ in the genome-wide meta-analysis to be included in the score. This allows us to explore whether variants that do not reach statistical significance in GWAS of current size contribute to disease susceptibility. We used the recommended significance threshold of *P* < 0.001 for determining significantly associated risk scores (38).

## Results

**Figure 1.**
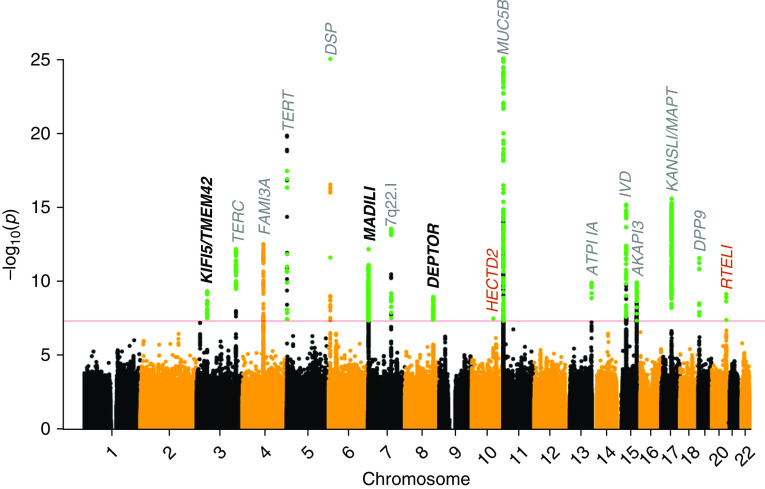
Manhattan plot of discovery analysis results. The *x* axis shows chromosomal position, and the *y* axis shows the −log(*P* value) for each variant in the discovery genome-wide analysis. The red line shows genome-wide significance (*P* < 5 × 10^−8^), and variants in green met the criteria for further study in the replication analysis (i.e., reached genome-wide significance in the discovery meta-analysis and had *P* < 0.05 and consistent direction of effects in each study). Genes in gray are previously reported signals that reach significance in the discovery genome-wide meta-analysis. Genes in black are the novel signals identified in the discovery analysis that reach genome-wide significance when meta-analyzing discovery and replication samples. The signals that did not replicate are shown in red. For ease of visualization the *y* axis has been truncated at 25.

Following quality control, 541 cases and 542 controls from the Chicago study, 1,515 cases and 4,683 controls from the Colorado study, and 612 cases and 3,366 controls from the UK study were available (Table 1 and Figure E1) to contribute to the discovery stage of the genome-wide susceptibility analysis (Figure 1). For the replication stage of the GWAS, after quality control, there were 792 cases and 10,000 controls available in the UUS study and 664 cases and 1,874 controls available in the Genentech study (*see* the online supplement).

**Table 1.** Demographics of Study Cohorts

	Chicago		Colorado		UK		UUS		Genentech	
	Cases	Controls	Cases	Controls	Cases	Controls	Cases	Controls	Cases	Controls
*n*	541	542	1,515	4,683	612	3,366	792	10,000	664	1,874
Genotyping array/sequencing	Affymetrix 6.0 SNP array		Illumina Human 660W Quad BeadChip		Affymetrix UK BiLEVE array	Affymetrix UK BiLEVE and UK Biobank arrays	Affymetrix UK Biobank and Spain Biobank arrays	Affymetrix UK BiLEVE and UK Biobank arrays	HiSeq X Ten platform (Illumina)	
Imputation panel	HRC		HRC		HRC		HRC		—	
Age, yr, mean	68	63*	66	—	70^†^	65	69	58	68	—
Sex, M, %	71^‡^	47^§^	68	49	70.8	70.0	75.2	72.1	73.5	27.1
Ever smokers, %	72	42	—	—	72.9^‖^	70.0	68.7^¶^	68.0	67.3	18.1**

*Definition of abbreviations*: HRC = Haplotype Reference Consortium; UUS = United States, United Kingdom, and Spain.

* Age only available for 103 Chicago controls.

^†^ Age available for 602 UK cases.

^‡^ Sex only available for 500 Chicago cases.

^§^ Sex only available for 510 Chicago controls.

^‖^ Smoking status only recorded for 236 UK cases.

^¶^ Smoking status only recorded for 753 idiopathic pulmonary fibrosis cases in UUS.

** Smoking status only recorded for 481 of the Genentech controls.

To identify new signals of association, we meta-analyzed the genome-wide association results for IPF susceptibility for the Chicago, Colorado, and UK discovery studies. This gave a maximum sample size of up to 2,668 cases and 8,591 controls for 10,790,934 well-imputed (*R*^2^ > 0.5) variants with minor allele count ≥10 in each study and which were available in two or more of the studies (Figure E2).

**Figure 2.**
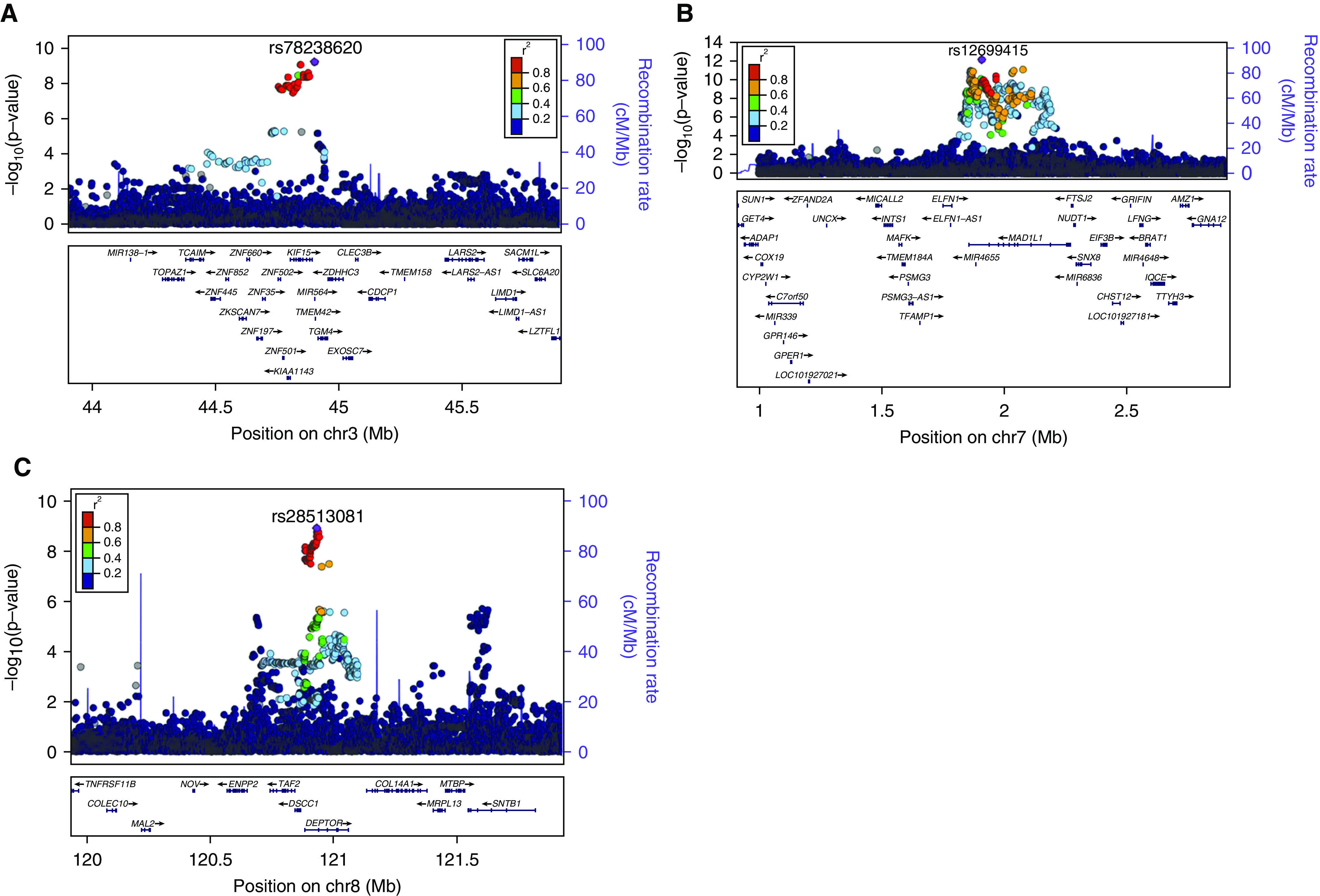
Region plots of three novel idiopathic pulmonary fibrosis susceptibility loci from discovery genome-wide meta-analysis. Each point represents a variant with chromosomal position on the *x* axis and the −log(*P* value) on the *y* axis. Variants are colored in by linkage disequilibrium with the sentinel variant. Blue lines show the recombination rate, and gene locations are shown at the bottom of the plot. Region plots are shown for the three replicated novel idiopathic pulmonary fibrosis susceptibility loci, i.e., (*A*) the susceptibility signal on chromosome 3 near *KIF15*, (*B*) the susceptibility signal on chromosome 7 near *MAD1L1*, and (*C*) the susceptibility signal on chromosome 8 near *DEPTOR*.

Three novel signals (in 3p21.31 [near *KIF15*, Figure 2A], 7p22.3 [near *MAD1L1*, Figure 2B], and 8q24.12 [near *DEPTOR*, Figure 2C]) showed a genome-wide significant (*P* < 5 × 10^−8^) association with IPF susceptibility in the discovery meta-analysis and were also significant after adjusting for multiple testing (*P* < 0.01) in the replication stage comprising 1,467 IPF cases and 11,874 controls (Tables 2 and E1). Two additional loci were genome-wide significant in the genome-wide discovery analysis but did not reach significance in the replication studies. The sentinel variants of these two signals were a low-frequency intronic variant in *RTEL1* (MAF = 2.1%, replication *P* = 0.012) and a rare intronic variant in *HECTD2* (MAF = 0.3%, replication *P* = 0.155). Conditional analyses did not identify any additional independent association signals at the new or previously reported IPF susceptibility loci (Figure E5).

**Table 2.** Discovery and Replication Association Analysis Results for the Five Signals Reaching Significance in the Discovery Genome-Wide Association Studies that Have Not Previously Been Reported as Associated with Idiopathic Pulmonary Fibrosis

Chr	Pos	rsid	Locus	Major Allele	Minor Allele	MAF (%)	Discovery Meta-Analysis		Replication Meta-Analysis		Meta-Analysis of Discovery and Replication	
							OR [95% CI]	*P* Value	OR [95% CI]	*P* Value	OR [95% CI]	*P* Value
3	44902386	rs78238620	*KIF15*	T	A	5.3	1.58 [1.37–1.83]	5.12 × 10^−10^	1.48 [1.24–1.77]	1.43 × 10^−5^	1.54 [1.38–1.73]	4.05 × 10^−14^
7	1909479	rs12699415	*MAD1L1*	G	A	42.0	1.28 [1.19–1.37]	7.15 × 10^−13^	1.29 [1.18–1.41]	2.27 × 10^−8^	1.28 [1.21–1.35]	9.38 × 10^−20^
8	120934126	rs28513081	*DEPTOR*	A	G	42.8	0.82 [0.76–0.87]	1.20 × 10^−9^	0.87 [0.80–0.95]	0.002	0.83 [0.79–0.88]	1.93 × 10^−11^
10	93271016	rs537322302	*HECTD2*	C	G	0.3	7.82 [3.77–16.2]	3.43 × 10^−8^	1.75 [0.81–3.78]	0.155	3.85 [2.27–6.54]	6.25 × 10^−7^
20	62324391	rs41308092	*RTEL1*	G	A	2.1	2.12 [1.67–2.69]	7.65 × 10^−10^	1.45 [1.08–1.94]	0.012	1.82 [1.51–2.19]	2.24 × 10^−10^

*Definition of abbreviations*: Chr = chromosome; CI = confidence interval; MAF = minor allele frequency; OR = odds ratio; Pos = position; rsid = reference SNP cluster ID.

The minor allele is the effect allele, and the MAF is taken from across the studies used in the discovery meta-analysis.

To identify the likely causal genes for each new signal, we investigated whether any of the variants were also associated with changes in gene expression (Table 3). The sentinel variant (rs78238620) of the novel signal on chromosome 3 was a low-frequency variant (MAF = 5%) in an intron of *KIF15* with the minor allele being associated with increased susceptibility to IPF and decreased expression of *KIF15* in brain tissue and the nearby gene *TMEM42* in thyroid (31) (Figure E7 and Tables E2 and E3i). The IPF risk allele for the novel chromosome 7 signal (rs12699415, MAF = 42%) was associated with decreased expression of *MAD1L1* in heart tissue (31) (Figure E8 and Tables E2 and E3ii). For the signal on chromosome 8, the sentinel variant (rs28513081) was located in an intron of *DEPTOR*, and the IPF risk allele was associated with decreased expression of *DEPTOR* (in colon, lung, and skin [27–29, 31]) and RP11-760H22.2 (in colon and lung [31]). The risk allele was also associated with increased expression of *DEPTOR* (in whole blood [30]), *TAF2* (in colon [31]), RP11-760H22.2 (in adipose [31]), and KB-1471A8.1 (in adipose and skin [31], Figure E9 and Tables E2 and E3iii). There were no variants predicted to be highly deleterious within the fine-mapped signals for any of the loci.

**Table 3.** Gene Expression and Spirometric Results for the Three Novel IPF Susceptibility Loci

Chr	rsid of Sentinel Variant	Annotation	eQTL		FEV_1_		FVC		FEV_1_/FVC	
			Lung Tissue	Nonlung Tissue	β [95% CI]	*P* Value	β [95% CI]	*P* Value	β [95% CI]	*P* Value
3	rs78238620	Intron (*KIF15*)	—	↓ *KIF15* ↓ *TMEM42*	−0.011 [−0.022 to 0.000]	0.069	−0.022 [−0.033 to 0.011]	2.92 × 10^−4^	0.017 [0.006 to 0.028]	0.005
7	rs12699415	Intron (*MAD1L1*)	—	↓ *MAD1L1*	−0.007 [−0.012 to −0.002]	0.011	−0.011 [−0.016 to −0.007]	1.41 × 10^−5^	0.008 [0.003 to 0.012]	0.005
8	rs28513081	Intron (*DEPTOR*)	↓ *DEPTOR* ↓ RP11-760H22.2	↕ *DEPTOR* ↕ RP11-760H22.2 ↑ KB-1471A8.1 ↑ *TAF2*	0.001 [−0.004 to 0.006]	0.822	−0.005 [−0.010 to −0.001]	0.045	0.011 [0.006 to 0.016]	4.22 × 10^−5^

*Definition of abbreviations*: Chr = chromosome; CI = confidence interval; eQTL = expression quantitative trait loci; IPF = idiopathic pulmonary fibrosis; rsid = reference SNP cluster ID.

Annotation of the variant was taken from Variant Effect Predictor (VEP). A list of all variants included in the credible sets with their annotations and eQTL results can be found in Table E3. For colocalization, only genes where there was a greater than 80% probability of colocalization between the IPF risk signal and gene expression of that gene are reported in this table. In the colocalization column, ↑ denotes that the allele that increases IPF risk was associated with increased expression of the gene, ↓ denotes that the IPF risk allele was associated with decreased expression of the gene, and ↕ denotes that the IPF risk allele was associated with increased expression in some tissues and decreased expression in others. Full results from the eQTL and colocalization analyses can be found in Table E2. The spirometric results for the three novel IPF risk loci are taken from Shrine and colleagues (36) using the allele associated with increased IPF risk as the effect allele, with β being the change in *z*-score units. Results for all IPF risk variants can be found in Table E6.

We confirmed genome-wide significant associations with IPF susceptibility for 11 of the 17 previously reported signals (in or near *TERC, TERT, DSP,* 7q22.1, *MUC5B, ATP11A, IVD, AKAP13, KANSL1, FAM13A*, and *DPP9*; Table E1 and Figure E4). The signal at *FAM13A,* while genome-wide significant in the discovery meta-analysis, was not significant in the Chicago study. This was the only signal reaching genome-wide significance in the discovery genome-wide meta-analysis that did not reach at least nominal significance in each study in the discovery analysis. Three further previously reported signals at 11p15.5 (near *MUC5B*) were no longer genome-wide significant after conditioning on the *MUC5B* promoter variant (Table E1), consistent with previous reports (6, 39).

Of the 14 IPF susceptibility signals (i.e., the 11 previously reported signals we confirmed and three novel signals), the only variant predicted to have a potential functional effect on gene regulation through disruption of chromatin structure or transcription factor binding motifs (using DeepSEA) was rs2013701 (in an intron of *FAM13A*), which was associated with a change in DNase I hypersensitivity in 18 cell types and FOXA1 in the T-47D cell line (a breast cancer cell line derived from a pleural effusion, Table E4). The 14 IPF susceptibility signals were found to be enriched in DNase I hypersensitivity site regions in multiple tissues including fetal lung tissue (Figures E10 and E11). No enrichment in differential expression in airway epithelial cells between IPF cases and healthy controls was observed for the 14 IPF susceptibility signals when using SNPsea (Table E5).

Previous studies have reported an overlap of genetic association loci between lung function and IPF (12). We undertook a lookup of the 14 IPF susceptibility loci in the largest GWAS of lung function in the general population published to date (36). The sentinel variants of 12 of the 14 IPF susceptibility loci were at least nominally associated (*P* < 0.05) with one or more lung function trait in general population studies (Tables 3 and E6). After adjustments for multiple testing (*P* < 5.2 × 10^−4^), the previously reported variants at *FAM13A*, *DSP*, and *IVD* were associated with decreased FVC, and variants at *FAM13A*, *DSP*, 7q22.1 (*ZKSCAN1*), and *ATP11A* were associated with increased FEV_1_/FVC. Similarly, for the three novel susceptibility variants, all showed at least a nominal association with decreased FVC and increased FEV_1_/FVC. We observed a nominally significant association of the *MUC5B* IPF risk allele with decreased FVC and increased FEV_1_/FVC. The IPF risk alleles at *MAPT* were significantly associated with both increased FEV_1_ and FVC. To determine how the variants identified for IPF susceptibility are related to differences in lung function between cases and controls, we investigated whether variants known to be associated with lung function show an association in our IPF GWAS. Of the 279 variants reported (36) as associated with lung function (Table E7), 8 showed an association with lung function after corrections for multiple testing (located in or near *MCL1*, *DSP*, *ZKSCAN1*, *OBFC1*, *IVD*, *MAPT*, and two signals in *FAM13A*).

As interstitial lung abnormalities may be a precursor to IPF in a subset of patients, and there have been previous reports of shared genetic etiology between IPF and ILAs (37, 40, 41), we investigated whether our three new signals and the 11 previously reported signals were associated with ILAs in the largest ILA GWAS reported to date (37). Eight of the IPF susceptibility loci were at least nominally significantly associated with either ILAs or subpleural ILAs with consistent direction of effects (i.e., the allele associated with increased IPF risk was also associated with increased ILA risk). The new *KIF15*, *MAD1L1*, and *DEPTOR* signals were not associated with ILAs (although the rare risk allele at *HECTD2* that did not replicate in our study showed some association with an increased risk of subpleural ILAs [*P* = 0.003] with a large effect size similar to that observed in the IPF discovery meta-analysis).

To quantify the impact of as yet unreported variants on IPF susceptibility, polygenic risk scores were calculated excluding the 14 IPF susceptibility variants (as well as all variants within 1 Mb). The polygenic risk score was significantly associated with increased IPF susceptibility despite exclusion of the known genetic association signals (including *MUC5B*). As the *P*_T_ for inclusion of variants in the score was increased, the risk score became more significant reaching a plateau at around *P*_T_ = 0.2 with risk score *P* < 3.08 × 10^−23^ and explaining around 2% of the phenotypic variation (Figure E12), suggesting that there is a modest but statistically significant contribution of additional as yet undetected variants to IPF susceptibility. Further increasing *P*_T_ beyond 0.2 did not improve the predictive accuracy of the risk score.

## Discussion

We undertook the largest GWAS of IPF susceptibility to date and identified three novel signals of association that implicated genes not previously known to be important in IPF.

The strongest evidence for the new signal on chromosome 8 implicates *DEPTOR*, which encodes the dishevelled, Egl-10 and Pleckstrin domain–containing mTOR-interacting protein. *DEPTOR* inhibits mTOR (mammalian target of rapamycin) kinase activity as part of both the mTORC1 and mTORC2 protein complexes. The IPF risk allele at this locus was associated with decreased gene expression of *DEPTOR* in lung tissue (Table E2). TGFβ-induced DEPTOR suppression can stimulate collagen synthesis (42), and the importance of mTORC1 signaling via 4E-BP1 for TGFβ-induced collagen synthesis has recently been demonstrated in fibrogenesis (43). *MAD1L1*, implicated by a new signal on chromosome 7 and eQTL analyses of nonlung tissue, is a mitotic checkpoint gene, mutations in which have been associated with multiple cancers including lung cancer (44, 45). Studies have shown that *MAD1*, a homolog of *MAD1L1*, can inhibit *TERT* activity (or possibly enforce expression of *TERT* when the promoter E-box is mutated) (45, 46). This could suggest that *MAD1L1* may increase IPF susceptibility through reduced telomerase activity. Another spindle-assembly–related gene (47), *KIF15,* was implicated by the new signal on chromosome 3 (along with *TMEM42*).

The genome-wide study also identified two signals that were not replicated after multiple testing adjustments. *RTEL1*, a gene involved in telomere elongation regulation, has not previously been identified in an IPF GWAS; however, the collective effect of rare variants in *RTEL1* has been reported as associated with IPF susceptibility (48–54). The ubiquitin E3 ligase encoded by *HECTD2* has been shown to have a proinflammatory role in the lung, and other *HECTD2* variants may be protective against acute respiratory distress syndrome (55). However, the lack of replication for these signals in our data suggests that further exploration of their relationship to interstitial lung diseases is warranted.

By combining the largest available GWAS datasets for IPF, we were able to confirm 11 of 17 previously reported signals. Conditional analysis at the 11p15.5 region indicated that previously reported signals at *MUC2* and *TOLLIP* were not independent of the association with the *MUC5B* promoter variant. Previously reported signals at *EHMT2*, *OBFC1*, and *MDGA2* were only found to be associated in one of the discovery studies and showed no evidence of an association with IPF susceptibility in the other two discovery studies. Only the 11 signals that we confirmed in our data were included in subsequent analyses.

The IPF susceptibility signals at *DSP, FAM13A*, 7q22.1 (*ZKSCAN1*), and 17q21.31 (*MAPT*) have also been reported as associated with COPD, although with opposite effects (i.e., the allele associated with increased risk of IPF being associated with decreased risk of COPD). Spirometric diagnosis of COPD was based on a reduced FEV_1_/FVC ratio. In an independent dataset of 400,102 individuals, eight of the IPF signals were associated with decreased FVC and with a comparatively weaker effect on FEV_1_. This is consistent with the lung function abnormalities associated with IPF, as well as the decreased risk of COPD. Of note, only around 3% of previously reported lung function signals (36) also showed association with IPF susceptibility in our study. This suggests that while some IPF susceptibility variants might represent genes and pathways that are important in general lung health, others are likely to represent more disease-specific processes.

Using polygenic risk scores, we demonstrated that, despite the relatively large proportion of disease susceptibility explained by the known genetic signals of association reported here, IPF is highly polygenic with potentially hundreds (or thousands) of as yet unidentified variants associated with disease susceptibility.

A strength of our study was the large sample size compared with previous GWAS and the availability of an independent replication dataset. A limitation of our study was that the controls used were generally younger in all studies included, and there were differences in sex and smoking distributions in some of the studies. As age, sex, and smoking status were not available for all individuals in four of our datasets, we were unable to adjust for these variables without substantially reducing our sample size. However, cases and controls in the UUS and UK datasets were matched for age, sex, and smoking. The three novel signals replicated in all of the discovery and replication datasets, providing reassurance that the signals we report are robust despite differences between the datasets. As we had limited information beyond IPF diagnosis status for a large proportion of the individuals included in the studies, we cannot rule out some association with other age-related conditions that are comorbid with IPF. However, other age-related conditions were not excluded from either the cases or controls. For the signals near *KIF15* and *MAD1L1*, there was substantial evidence for an association with gene expression in nonlung tissues but not in either of the two (nonfibrotic) lung tissue eQTL datasets. This could reflect cell type-specific effects that are missed when studying whole tissue or effects that are disease-dependent. Finally, our study was not designed to identify rare functional variant associations. As both common and rare variants are known to be important in IPF susceptibility (39), this is a limitation of our study.

In summary, we report new biological insights into IPF susceptibility and demonstrate that further studies to identify the genetic determinants of IPF susceptibility are needed. Our new signals of association with IPF susceptibility provide increased support for the importance of mTOR signaling in pulmonary fibrosis as well as the possible implication of mitotic spindle-assembly genes.
